# Abnormal Breathing Patterns Predict Extubation Failure in Neurocritically Ill Patients

**DOI:** 10.1155/2017/9109054

**Published:** 2017-02-28

**Authors:** Pragya Punj, Premkumar Nattanmai, Pravin George, Christopher R. Newey

**Affiliations:** ^1^Department of Neurology, University of Missouri, 5 Hospital Drive, CE 540, Columbia, MO 65211, USA; ^2^Cleveland Clinic, Cerebrovascular Center, Neurological Institute, 9500 Euclid Avenue, Cleveland, OH 44195-5245, USA

## Abstract

In neurologically injured patients, predictors for extubation success are not well defined. Abnormal breathing patterns may result from the underlying neurological injury. We present three patients with abnormal breathing patterns highlighting failure of successful extubation as a result of these neurologically driven breathing patterns. Recognizing abnormal breathing patterns may be predictive of extubation failure and thus need to be considered as part of extubation readiness.

## 1. Introduction

Extubation in neurologically injured patients can be challenging. Typical factors considered for extubation readiness are stable cardiorespiratory status along with decreased risk for aspiration [1, 2]. Common predictors of extubation failure are longer duration on mechanical ventilation, poor cough reflex, upper airway obstruction, low hemoglobin concentration, continued intravenous sedation, and comorbid medical conditions such as left ventricular failure [1, 2]. Despite optimizing these components, extubation failure still occurs in 2 to 25% of cases in the intensive care unit (ICU) likely from a disparity between work of breathing (WOB) and capacity of respiratory muscles [1, 2]. A trial of spontaneous breathing (SBT) is typically performed prior to extubation as a predictor of success [1, 3]. If a patient breathes shallow and rapidly during the trial, the patient may not tolerate spontaneous breathing [1, 3]. However, neurologically injured patients have abnormal breathing patterns as a result of their injury. These patients may breathe rapid and/or irregularly as a result of their injury. Thus, SBTs are less reliable. Predictors of extubation success in these patients are less clear. This article describes three cases of extubation failure who had an associated abnormal breathing pattern as a result of their underlying neurological injury.

## 2. Case Report

### 2.1. Case 1

**Figure 1:**
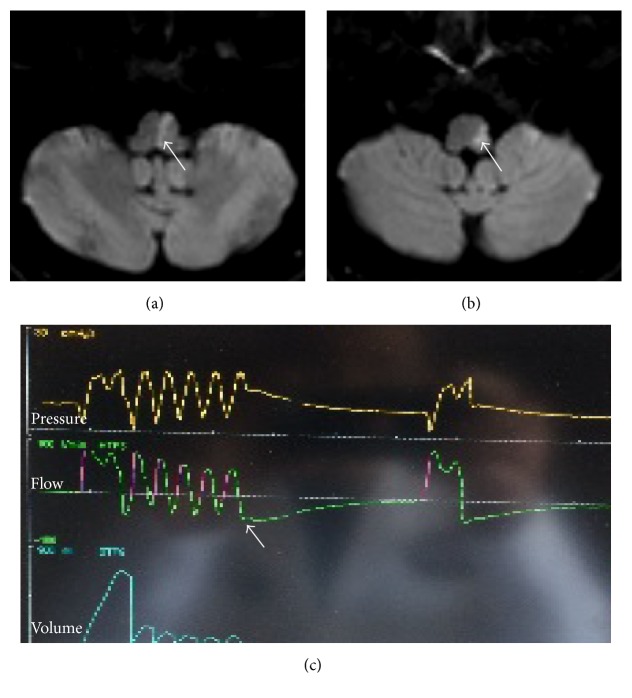
Magnetic resonance imaging of the brain. Diffusion weighted imaging (DWI) showing left medial (a, arrow) and lateral (b, arrow) medullary acute ischemia. (c) Ventilator picture showing cluster breathing pattern (arrow).

An 80-year-male presented to the emergency department (ED) with acute onset of right sided facial droop, right sided hemiplegia, numbness, swallowing difficulty, and dysphonia. He had a past history of hypertension, diabetes, gout, prior ischemic stroke without any residual neurological deficit, and peripheral vascular disease. On examination, he was noted to have nystagmus on upward gaze and weakness with loss of sensation in the right upper and lower extremities. He was unable to coordinate the control of secretions. He was intubated for airway protection given his poor orolingual control. A magnetic resonance imaging (MRI) of the brain was obtained which showed left lateral and medial medullary acute ischemia (Figures 1(a)-1(b)).

His hospital stay was complicated by aspiration pneumonia. He was treated with antibiotics. Subsequently, his chest X-ray and neurological examination improved. However, the waveform on the ventilator showed a cluster breathing pattern (Figure 1(c)). He was eventually extubated. However, he did not tolerate secondary breathing irregularity and was subsequently reintubated. A tracheostomy and percutaneous gastrostomy were performed.

### 2.2. Case 2

**Figure 2:**
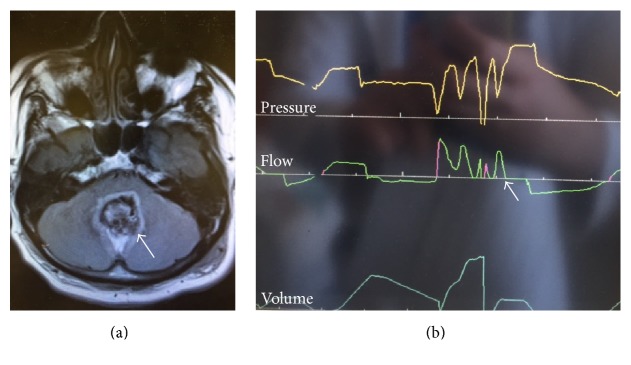
Magnetic resonance imaging of the brain. (a) T2 fluid attenuated inversion recovery (FLAIR) sequence showing medulloblastoma status after resection causing effacement of the fourth ventricle (arrow). (b) Ventilator picture showing cluster breathing pattern (arrow).

A 60-year-male presented to the ED with headache, frequent falls, and dizziness for prior 3 months. He had a past medical history of glaucoma and hypothyroidism. Computed tomography (CT) head showed a large medial cerebellar mass with compression of the brainstem and effacement of the fourth ventricle. The mass was resected (Figure 2(a), arrow). Postoperatively, he remained intubated for stuporous mental state.

His hospital course was complicated by aspiration pneumonia and development of acute respiratory distress syndrome (ARDS). He made remarkable recovery from the lung injury. His level of consciousness remained lethargic. The ventilator waveform consistently showed cluster breathing pattern preventing extubation (Figure 2(b), arrow). A tracheostomy and percutaneous gastrostomy were then performed.

### 2.3. Case 3

**Figure 3:**
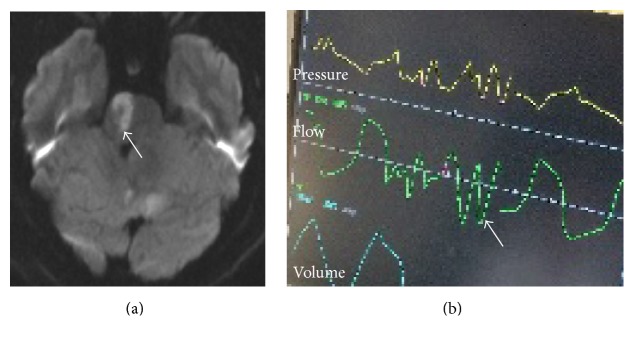
Magnetic resonance imaging of the brain. (a) Diffusion weighted imaging (DWI) showing right acute pontine ischemia (arrow). (b) Ventilator picture showing ataxic breathing pattern (arrow).

A 43-year-old male presented to the ED with altered mental status, slurred speech, and left sided hemiplegia. He had a past medical history of diabetes, hypertension, and hypertriglyceridemia. Neurological examination confirmed the left sided hemiplegia and decreased arousal. CT head showed moderate sized left medial posterior inferior cerebellar artery (PICA) infarct with obstructive hydrocephalus. MRI confirmed the CT scan findings (Figure 3(a), arrow). CT-angiography (CTA) demonstrated right vertebral artery dissection. His examination acutely worsened. He was intubated and required a decompressive suboccipital craniectomy. He slowly improved and eventually was extubated. He failed extubation and was reintubated. The ventilator waveform on reintubation showed an ataxic breathing pattern (Figure 3(b), arrow). He eventually required tracheostomy and percutaneous gastrostomy.

### 2.4. Weaning

Prior to extubation, a standard checklist was reviewed. The patients' respiratory statuses were PaO2 > 60 mmHg with FiO2 < 50% and positive end expiratory pressure (PEEP) of <8. The patients were hemodynamically stable. Arterial blood gasses showed a pH range of 7.44–7.46, PaO2 68–98 mmHg, PaCO2 of 33–35 mmHg, and bicarbonate of 23-24 mEq/L. Spontaneous breathing trials were performed in all three patients. This breathing trial consisted of a 30-minute trial on 0 pressure support and 0 PEEP. All three patients passed this breathing trial. Patient 2 was not extubated given his lethargic mental status and cluster breathing pattern. The other two patients' levels of consciousness were awake and following commands. All three patients had a cough reflex.

## 3. Discussion

We presented three cases of neurologically injured patients who were otherwise ready for extubation. However, they each had a neurologically driven breathing pattern. All required tracheostomy and percutaneous gastrostomy. These cases highlight that the finding of a breathing abnormality attributed to the underlying brain injury should be considered when predicting extubation success.

The patients of these three cases were unable to be extubated secondary to abnormal neurogenic breathing patterns. Cluster breathing pattern, as observed in cases 1 and 2, has been characterized as “respiratory pattern characterized by periods or clusters of rapid respirations of near equal depth or tidal volume followed by regular periods of apnea” [4]. It classically results from injury to pons due to stroke, trauma, or increased pressure on medulla from tumor or herniation [4]. Ataxic breathing as seen in case 3 is classically seen in medullary ischemia [4, 5]. However, as we demonstrate here, the breathing patterns may be seen in injuries elsewhere in the brainstem. For example, cluster breathing, as in case 1, associated with medullary ischemia, and ataxic breathing, as in case 3, associated with pontine ischemia.

The respiratory system functions to maintain adequate oxygenation for energy production and ventilation to maintain physiological acid base balance [6]. The primitive respiratory control centers are located within the pons and medulla of the brainstem [6]. It receives afferent signals from peripheral and central chemoreceptors, stretch receptors in lung, and higher cortical centers [6]. These afferent inputs are analyzed and efferent signals are sent to respiratory muscle groups to maintain a stable tidal volume as well as respiratory rate and rhythm [6]. Damage to any of these areas can create classic respiratory patterns [5, 6]. These include Cheyne Stokes (bilateral cerebral hemisphere injury), hyperventilation (midbrain injury), apneustic (cephalad pons injury), cluster (caudal pons/cephalad medulla injury), and ataxic (medullary injury) breathing patterns [5, 6]. These breathing patterns are summarized in Table 1.

The decision to perform tracheostomy in the neurocritical care patient is individualized. Predictors for tracheostomy in patients with intracerebral hemorrhage have been proposed to be hematoma volume (≥30 cc), intraventricular hemorrhage, obstructive hydrocephalus, Glasgow Coma Scale (GCS; <8), prolonged intubation (*≥*14 days), and pneumonia [7]. The timing of when to perform tracheostomy (early or late) in intracerebral hemorrhage patients has been proposed with the TRACH score [8]. Significant predictors from the TRACH score indicating need for early tracheostomy included GCS score and radiological findings of obstructive hydrocephalus, septum pellucidum shift, and thalamic hemorrhage [8]. Early tracheostomy may be beneficial in the neurocritical care patient. In subarachnoid hemorrhage patients, performing early (days 1–7) versus later (days 8–20) tracheostomy resulted in less pneumonia, less respiratory adverse events, shorter duration of mechanical ventilation, and ultimately earlier decannulation [9]. In an analysis of 10 trials in acute brain injured patients, early tracheostomy was found to reduce long-term mortality, duration of mechanical ventilation, and ICU length of stay; however delaying tracheostomy resulted in an increased probability of not needing a tracheostomy [10]. Assessing need for tracheostomy at day 7 has been recommended [11]. However, in general critical care, early tracheostomy only reduced the duration of ventilator but not the incidence of pneumonia, length of stay, or long-term mortality [12]. Another study in critically ill patients only found early tracheostomy useful in reducing duration of sedation [13]. It was not found to change mortality, pneumonia incidence, duration of mechanical ventilation, or length of stay [13]. In general, we assess the need for tracheostomy around day 7. Unfortunately, algorithms for determining who would benefit from tracheostomy and timing of tracheostomy are largely lacking and do not include neurologically driven breathing patterns.

In conclusion, the finding of neurologically driven breathing patterns should be considered in predicting extubation failure.

## Figures and Tables

**Table 1:** Neurologically driven breathing patterns and anatomic correlation.

Respiratory control centers	Function	Abnormal breathing pattern associated with injury
Cerebral hemisphere	Regulates respiratory center located in pons and medulla	Cheyne stokes breathing pattern
Midbrain	Rate of inspiration	Hyperventilation
Pons		
Pneumotaxic center	Intensity of respiration	Cluster breathing pattern
Apneustic center	Rate of inspiration	Apneustic breathing pattern
Medulla oblongata		
Ventral breathing center	Responsible for expiratory movement	Ataxic breathing pattern
Dorsal breathing center		
